# Emergency Caesarean Section in a COVID-19 Infected Mother in a Primary Health Care Centre

**DOI:** 10.31729/jnma.7450

**Published:** 2022-05-31

**Authors:** Anil KC, Prativa Subedi, Sujata Dahal, Ujjawal Poudel

**Affiliations:** 1 Burtibang Primary Health Centre, Dhorpatan-1, Baglung, Nepal; 2 Rolpa Hospital, Khalanga, Rolpa, Nepal; 3 Patan Academy of Health Sciences, Lagankhel, Patan, Nepal; 4 Jibjibe Rural Hospital, Jibjibe, Rasuwa, Nepal

**Keywords:** *caesarean section*, *COVID-19*, *health equity*, *Nepal*, *primary health care*

## Abstract

People from rural areas of Nepal struggle to have access to adequate medical care on time. Most of the tertiary centres are overburdened by patients, while the peripheral health facilities have been unable to function efficiently due to a lack of infrastructures and skilled manpower needed to run hospitals smoothly. We present a case of a 21-year-old primigravida at 41 weeks and 3 days of gestation with mild COVID-19 symptoms who underwent a Caesarean section for non-progression of labour and foetal distress at a primary health care centre in Nepal; however, both maternal and foetal outcomes were favourable. Therefore, upgrading the quality of care in peripheral health facilities can help in the achievement of accessibility, equity, and quality in health care service in Nepal.

## INTRODUCTION

With the emphasis of the Safe Motherhood Program in Nepal, there has been a significant increase in institutional deliveries, resulting in a significant decrease in maternal and newborn mortality rates over the past few years. But coronavirus disease pandemic jeopardised maternal and neonatal health. It took longer to seek, get to and receive health care because of the fear of contracting a virus and the imposition of mobility limitations.^1^ Inadequate antenatal visits raised the number of high-risk pregnancies and emergency deliveries.^2,3^ Providing emergency obstetric care in a low-resource setting is a challenging job. Here we present a short communication of a successful Caesarean section performed on a COVID-19 infected mother in a resource-limited setting.

## Case Study

A 21-year-old primigravida presented to Burtibang primary health centre at 41 weeks and 3 days of gestation on 4^th^ July, 2021. She had a history of fever and cough for 3 days, with a loss of taste sensation that began 1 day after the fever and cough. At the time of presentation, her vitals were stable with a blood pressure of 100/70 mm Hg, pulse rate of 80 beats per minute, a temperature of 37°C, respiratory rate of 20 breaths per minute, and saturation was 96% in room air. On obstetric examination, the foetus had a longitudinal lie with cephalic presentation. The head was engaged with moderate contractions.

The foetal heart rate was 142 beats per minute and regular. On per vaginal examination, a cervical opening was 3 cm dilated with 30% effacement. The head station was at -2 and the membrane was intact. Because of her suspected symptoms, a COVID-19 antigen test was performed using a nasopharyngeal swab which revealed she had an infection. The patient was admitted to an isolation ward and closely monitored for progression of labour. Oxytocin was used for the augmentation of labour; however, subsequent partographs showed poor progression of labour. After 12 hours of admission, the cervical os was only 5 cm dilated and the foetal heart rate declined to 110 beats per minute. As a result of failed augmentation and foetal bradycardia Caesarean section was warranted.

We informed the patient party that there was a risk to the mother and the baby owing to the lack of a paediatrician, anesthesiologist, blood bank, and critical care service at our facility. However, even the closest referral centre Dhaulagiri zonal hospital was at 5 hours travel distance by ambulance and the nearest hospital with Intensive Care Unit (ICU) service was 6 hours drive. Furthermore, due to financial constraints, the patient party was unable to afford the cost of the tertiary care centre. Thus, a Caesarean section was planned in the primary health centre with the consent of the husband and patient.

The patient's pre-operative investigations were within normal limits. Following signing the highrisk informed written consent form, the patient was shifted to the operation theatre. A Caesarean section was performed under spinal anaesthesia. All of the health care professionals involved in the surgery were equipped with full Personal Protective Equipment (PPE) including a gown, surgical gloves, N95 mask, and boots. The intraoperative period was uneventful. A single, alive, male baby was born with a birth weight of 3000 grams and with Apgar scores of 7/10, 8/10, and 9/10. The rapid antigen test of the baby was done 12 hours after birth which was negative.

Mother was admitted to the primary health centre with intravenous antibiotics injection of ceftriaxone, injection of gentamicin, and injection of metronidazole and analgesics. Except for a temperature of 38.89°C on the first postoperative day, she remained afebrile throughout her postoperative period.

However, on a postoperative day, her cough started to worsen and saturation dropped to 85%. Patient parties were 3rd about the declining oxygen saturation level and advised for referral but they refused once more due to financial constraints. She was thus provided oxygen via nasal prong at 2 litres per minute. Her oxygen saturation started to improve and became normal on the 5^th^ postoperative day. Despite the baby developing a mild cough on the 2^nd^ day, his saturation was normal. A real-time polymerase chain reaction test was performed on the newborn and all of the health personnel involved in the operation; all of the results were negative. She was discharged on the 7^th^ postoperative day after suture removal.

## DISCUSSION

The aforementioned case pertains to the first wave of COVID-19 where there was sheer fear of the virus and hospitals were reluctant to take the risk of treating COVID-19 positive individuals. Caesarean section of a COVID-19 positive mother was performed in Burtibang primary health centre even before it was done in the Dhaulagiri zonal hospital (a referral centre). The operating doctor and his team took the risk and followed all the standard precautions; fortunately, the outcome was favourable. Nevertheless, this might not always be the case. It can be very challenging to provide routine services, let alone perform surgeries in low-resource settings due to the unavailability of trained human resources and essential infrastructure and equipment.

Spinal anaesthesia might fail or patients might develop anaesthesia-related complications that can be hard to manage even in a tertiary hospital.^4^ The absence of a blood bank in the fate of postpartum haemorrhage can be fatal for mothers. It is possible that inadequate neonatal resuscitation could lead to encephalopathy or even death.^5^ While these constitute general complications, in the case of a COVID-19 patient, the lack of knowledge of disinfection, donning, and doffing among the staff could rather increase the chance of spreading of the virus within the health facility.^6,7^ Moreover, the increasing incidence of violence against health professionals and vandalism in health facilities has made doctors more hesitant to take the risk of treating patients.^8^ Having said that, if our health system continues to rely solely on service delivery by tertiary hospitals, the health status of Nepalese people can never be uplifted.

According to a study conducted in three hilly districts of Nepal, out of 33 maternal deaths, 13 were due to the cost of transport service/health facility, eight were due to lack of transport service, and four were due to lack of health facility.^9^ An important barrier to timely referral is the frail economy of rural inhabitants of Nepal. A study done in the district hospital of rural Nepal showed that the direct and indirect cost of a patient referred per surgery was US$80, which was over 1.5 times the local district's per capita income.^10^ These studies' data highlight the need to upgrade peripheral health facilities to decrease the need of a referral.

## WAY FORWARD

It is very crucial to focus on the capacity building of health professionals and infrastructure development in peripheral health facilities to meet the goals of universal health coverage in Nepal. The difficult topography and geographic constraints increase both the cost and time for transport to a higher facility. If peripheral health facilities are strengthened in terms of human and physical resources, it also decreases the burden of patients in the central and tertiary centres, thereby improving the overall quality of health care in the country. With the introduction of the federal framework in the constitution, it is important that all stakeholders work for the decentralisation of plans, policies, and actions for health governance and reforms in Nepal.
